# Important Correction

**Published:** 1858-01

**Authors:** D. Brainard

**Affiliations:** Chicago


### EDITORIAL.

### IMPORTANT CORRECTION.

CHICAGO, Jan. 7, 1858.

TO THE EDITORS OF THE CHICAGO MEDICAL JOURNAL:

GENTLEMEN,—My attention has been called to an article published in the *Peninsular Medical Journal* some months since, making an attack upon my character, professionally and otherwise, on account of testimony alleged to have been given by me in a case before a court in this city, relating to the subject of criminal abortion.

So long as the publication was confined to the journal in question it was unworthy of notice, the well known proclivity of some of its editors to libelous attacks upon members of the profession being sufficiently understood in the region where it circulates. The anonymous character of the original attack, its being in a political newspaper, written by a non-professional man, or a member of the profession who concealed his name, ought, in my judgment, to have prevented respectable journalists from copying or giving it currency without taking pains to ascertain its truth or falsity. Having learned that some editors of medical journals, of whom a different course might reasonably be expected, have copied the article in question, I beg you will be kind enough to say that the report of the testimony in the case referred to, in so far as I am concerned, is essentially false and garbled.

By so doing, you will oblige your obedient servant,

D. BRAINARD.

[We would call the attention of the journals who have copied the attack above referred to, to this statement of Dr. Brainard in regard to the facts of the case.—EDS.]

